# Risk-taking in traffic is associated with unhealthy lifestyle: Contribution of aggressiveness and the serotonin transporter genotype

**DOI:** 10.1016/j.nsa.2022.100110

**Published:** 2022-09-21

**Authors:** Tõnis Tokko, Diva Eensoo, Kadi Luht-Kallas, Jaanus Harro

**Affiliations:** a Department of Psychology, University of Tartu, Estonia; b Department of Chronic Diseases, Research Centre, National Institute for Health Development, Tallinn, Estonia; c Rescue College, Estonian Academy of Security Sciences, Tallinn, Estonia; d Department of Neuropsychopharmacology, Institute of Chemistry, University of Tartu, Estonia

**Keywords:** Traffic behavior, Impulsivity, Aggressiveness, 5HTTLPR, Food choice, Workout

## Abstract

**Objectives:**

Risk taking behaviour, including in traffic, is related to impulsivity and aggressiveness, and so is unhealthy lifestyle. The serotonin transporter gene promoter polymorphism (5-HTTLPR) has been associated with impulsivity, aggression, alcohol use, speed limit exceeding and traffic accidents. The aim of this study was to examine whether subjects with less healthy eating and exercise habits take more risks in traffic, and whether impulsivity, aggressiveness and the serotonin transporter genotype could mediate or moderate any such associations.

**Method:**

A sub-sample of the Estonian Psychobiological Study of Traffic Behaviour (EPSTB (n ​= ​817) with mean age (SD) ​= ​31.4 (10.0) years filled out lifestyle questionnaires. Impulsivity was measured by Adaptive and Maladaptive Impulsivity Scale and aggressiveness by Buss – Perry Aggression Questionnaire. Traffic violation data in the previous 5 years period were obtained from police database.

**Results:**

Speed limit exceeders had higher physical and verbal aggression, higher AUDIT scores, they reported more vigorous physical activity and drinking energy drinks more often. Path analysis showed that higher AUDIT scores were associated with speeding via higher physical aggression. 5-HTTLPR was not directly associated with speeding or driving while impaired by alcohol (DWI), but 5-HTTLPR s’-allele carriers had lower AUDIT scores if they were not junk food eaters and the other way around, while l’/l’ homozygosity was associated with DWI via higher AUDIT scores.

**Conclusion:**

Significant associations exist between risky traffic behaviour and aspects of lifestyle such as consumption of alcohol or junk food or energy drinks, as well as engagement in vigorous physical activity, while traits such as aggressiveness and the variation in the serotonergic system appear as mediating and moderating factors. Interventions preventing accidents should focus on wider array of behaviours and use personalized approach. Genetic variation should be investigated regarding associations with risk taking and health behaviour, and response to interventions.

## Highlights

• Speed limit exceeders had higher physical and verbal aggression.
• Consumption of energy drinks often was associated with taking risks in traffic.
• Speeding subjects reported more vigorous physical activity.
• Associations between lifestyle and speeding are mediated/moderated by 5-HTTLPR.

## 1 Introduction

Road traffic injuries remain a serious public health issue. Traffic accidents often result from risk taking behaviour that is based on behavioural traits such as impulsivity and aggressiveness (Constantinou et al., 2011; Biçaksiz and Özkan, 2016; Antić et al., 2018). Impulsive and aggressive behaviour have also been found associated with health behaviours like exercising and maintaining a healthy diet. For example, impulsivity has consistently been found related to not only overeating and food addiction (Loxton, 2018) but also to greater fast food consumption (Garza et al., 2016). Fast food/junk food is any food, which is low in essential nutrients and high in everything else - in particular, calories and sodium (Segen's Medical Dictionary, 2022). Consumption of energy drinks, which contain high amounts of caffeine and sugar, is another example of questionable diet: Associations have been found between energy drink consumption and higher perceived stress, smoking and alcohol abuse, poor quality of sleep, increased blood pressure, and risk of obesity and type 2 diabetes (Ali et al., 2015; Al-Shaar et al., 2017; Gunja and Brown, 2012; Wolk et al., 2012). Among possible mediating mechanisms, aggressive behaviour and sensation seeking are both associated with energy drinks consumption (Azagba et al., 2014; Hamilton et al., 2013). While physical exercise brings about multiple benefits to health, problematic practice of physical exercise (PPPE, also known as exercise addiction or exercise dependence) is a maladaptive pattern of excessive exercise behaviour that manifests in physiological, psychosocial, and cognitive symptoms (Hausenblas and Downs, 2002). Associations of PPPE with negative urgency and sensation seeking have also been found, and it has been suggested that PPPE serves to regulate or alleviate negative affect or aversive emotional states (Kotbagi et al., 2017).

Expectedly, attention deficit hyperactivity disorder (ADHD), with one of the core symptom domains being hyperactivity/impulsivity (DSM 5), has also been associated with traffic accidents and violations (Kittel-Schneider et al., 2019; Vaa, 2014), and we have recently found in independent samples that while approximately 12% of drivers have high ADHD risk by the ASRS screening score (Kessler et al., 2005), they also have more of recorded traffic accidents and violations (Tokko et al., 2022). Furthermore, people with ADHD symptoms also have higher rates of self-reported unhealthy lifestyles (e.g., alcohol/drug abuse, smoking, poor diet), and worse general health (Björk et al., 2018; Weissenberger et al., 2018). Lifestyles like these can result in different health problems (obesity, hypertension, heart disease), in addition to risky behaviour (Weissenberger et al., 2018).

Animal studies strongly suggest that poor dietary choices may influence neurodevelopmental trajectories during adolescence (Reichelt and Rank, 2017). More specifically, alterations in dopamine-mediated reward signalling and GABA-ergic inhibitory neurotransmission can occur and predispose individuals to dysregulated eating and impulsive behaviours Diets high in processed fat and sugar induce impulsive choice behaviour (Steele et al., 2017). Evidence also indicates that the relationship between somatic markers (body fat percentage, insulin, and inflammation) and impulsive choice is indeed moderated by diet, and the combination of such bodily measures and diet is most predictive of an impulsive choice (Steele, 2019).

Impulsive behaviour has most consistently been associated with low capacity of the central serotonergic system (Evenden, 1999; Fairbanks et al., 2001), and the serotonin transporter plays the crucial role in serotonergic neurotransmission. The serotonin transporter gene promoter polymorphism (5-HTTLPR) (Heils et al., 1995) has been associated with impulsivity (Steiger et al., 2005; Paaver et al., 2008), aggression (Gerra et al., 2005; Gonda et al., 2009), alcohol use (Merenäkk et al., 2011; Vaht et al., 2014; Eduardo Coral de Oliveira et al., 2016), suicide (Gonda et al., 2010) speed limit exceeding and traffic accidents (Eensoo et al., 2018). Some evidence exists that diets poor in the serotonin precursor tryptophan may induce depression (Shabbir et al., 2013), and acute tryptophan depletion has been shown to increase impulsivity in males (Walderhaug et al., 2002; Dougherty et al., 2010). The short (s) allele of the serotonin transporter gene promoter region polymorphism (5-HTTLPR) has been found to be a marker of less efficient serotonergic functioning, but being a common variant, it is not surprising that besides the risk behaviours it also confers higher adaptivity to the environment (Homberg and Lesch, 2011). Tryptophan depletion and the s-allele of 5-HTTLPR have been reported to be independently and additively associated with impulsivity (Walderhaug et al., 2010). We have previously shown that the 5-HTTLPR s’-allele carriers have less violations and accidents in traffic, compared to l’/l’ homozygotes (Eensoo et al., 2018), and lower adaptive impulsivity (Luht et al., 2019). It should however be noted that different markers of serotonergic capacity associate with the impulsivity profiles in e.g., drunk drivers vs speed limit exceeders (Paaver et al., 2006).

**Fig. 1:**
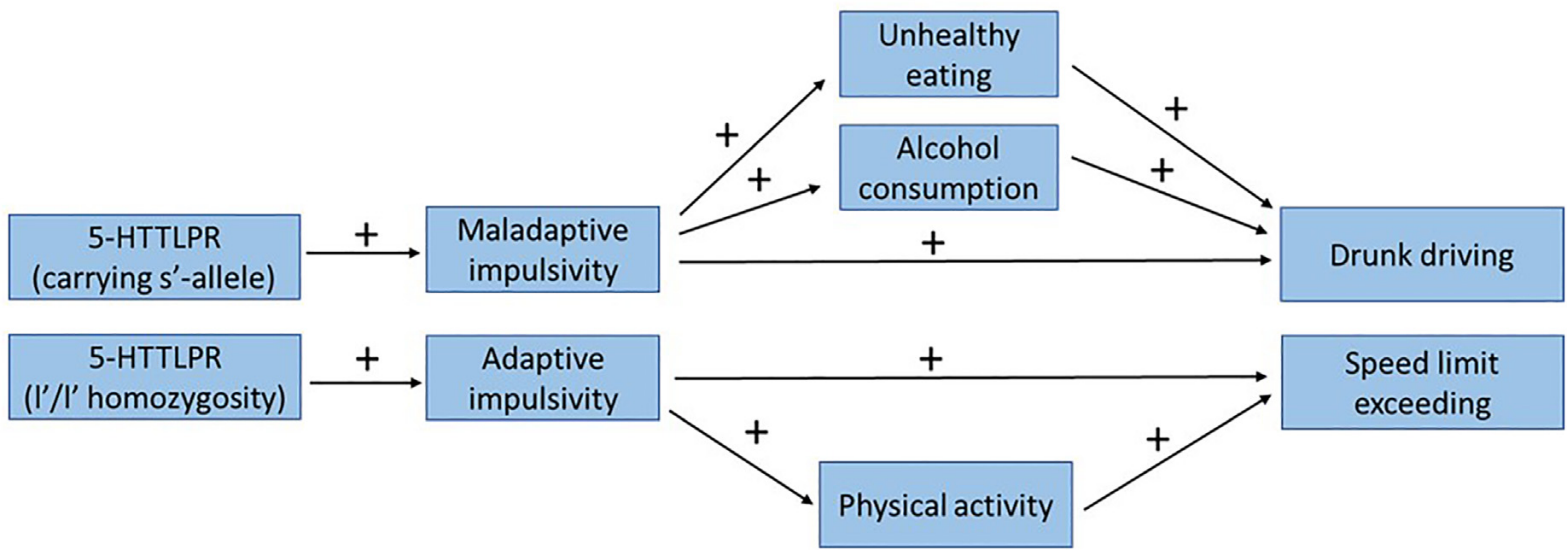
Schematic representation of the hypothesis of the study.

The aim of this study was to test the hypothesis that higher impulsivity leads to both less healthy lifestyle and taking risks while driving a car in traffic, the latter resulting in traffic violations and/or accidents. We also examined the possibility that the 5-HTTLPR polymorphism underlies the eventual associations (Fig. 1).

## 2 Method

### 2.1 Sample

A link to the web-based questionnaire was sent by e-mail in 2019 to all subjects of the Estonian Psychobiological Study of Traffic Behaviour (EPSTB). EPSTB comprises several samples recruited in four originally independent projects: Samples of drunk drivers (Eensoo et al., 2005) and speed limit exceeders (Paaver et al., 2006) with driver's license registry based controls, and two samples of traffic school students (Luht et al., 2019; Paaver et al., 2013). In brief, these samples had originally been formed as follows: The samples drunk drivers (Eensoo et al., 2005) and speed limit exceeders (Paaver et al., 2006) were formed of the male subjects from the police database of driving violations and control groups were formed of male subjects with a valid driving licence in the driving licence database by computerised random choice. Controls were screened for any police records. Two samples of traffic school students (Luht et al., 2019; Paaver et al., 2013) comprised driving school students applying for a passenger car driving license. All of the subjects who filled out the questionnaires were included in the current study. Altogether 817 subjects agreed to participate, with the mean age (SD) ​= ​31.4 (10.0) years); 49.2% males and 50.8% females. For this study subjects' age was calculated by adding 5 years to their age as it was in the beginning of their study, because we have information from databases about subjects' accidents and violations for a 5-year period since the beginning of study for each sample. The proportions of subjects by the year their study started were as follows: 2001, n ​= ​183 (22%); 2007, n ​= ​285 (35%); 2013/2014, n ​= ​349 (43%).

### 2.2 Questionnaires

#### 2.2.1 Impulsivity measures

The Adaptive and Maladaptive Impulsivity Scale (AMIS) was used to measure different facets of impulsivity (fast decision making, thoughtlessness, disinhibition, excitement seeking) as previously described (Laas et al., 2010). AMIS is based on the concept of functional and dysfunctional impulsivity by Dickman (1990). Subjects were asked to assess how much the 24 impulsivity-related statements applied to them on a scale from 1 to 5. Questionnaire was filled out by subjects in the beginning of the study and data were available for 774 subjects.

#### 2.2.2 Aggressiveness and driving anger

The Buss–Perry Aggression Questionnaire (BPAQ) (Buss and Perry, 1992) is a widely used measure of aggressiveness. The 29-item instrument assesses four aspects of aggressive behaviour: Physical aggression, Verbal aggression, Anger, and Hostility. Participants rated each statement on a 5-point Likert Scale (uncharacteristic ​= ​1, characteristic ​= ​5). Buss-Perry Aggression Questionnaire was filled out by all subjects in 2019, and data were available for 744 subjects.

The Driving Anger Scale (DAS 33) (Deffenbacher et al., 1994) includes 33 potentially angering traffic situations and queries how much anger does each provoke on a scale from 0 to 4 (0 ​= ​none at all, 1 ​= ​a little, 2 ​= ​some, 3 ​= ​much, 4 ​= ​very much). Driving Anger Scale was filled out by subjects in the beginning of the study and data were available for 568 subjects.

#### 2.2.3 Eating questionnaire

Subjects answered questions about the frequency (1–7, never – several times in a day) of eating different foods and drinking energy drinks in the past few weeks. We calculated the used the mean score of eating of French fries and hamburgers as an indicator of fast/unhealthy eating (junk food consumption, n ​= ​816). As an indicator of healthy eating, we used the question about the frequency of eating vegetables (n ​= ​806). In addition, we included the frequency of energy drink consumption (at least once a week vs less than once a week; n ​= ​816) in the analysis. Data were available for all subjects included in the analysis.

#### 2.2.4 Physical activity and body mass index

Subjects were asked about their physical activity in the past week. In this study we used “vigorous physical activity” as a measure. Vigorous physical activity was defined as activities that require great physical energy and make the subject breathe a lot faster than regularly (e.g., doing hard work, exercising). The activity had to last at least 10 ​min consecutively, and it was asked how much time the participants spent in the past week on these kinds of activities (hours per week). In addition, body mass index (BMI) was calculated by data provided by subjects about their weight and height (BMI ​= ​kg/m^2^). Data about physical activity were available for 812 and about BMI for 777 subjects.

#### 2.2.5 Alcohol consumption (AUDIT)

The Alcohol Use Disorders Identification Test (AUDIT) (Saunders et al., 1993) was used to identify subjects who have problematic alcohol consumption. The test contains 10 multiple choice questions on quantity and frequency of alcohol consumption, drinking behaviour, and alcohol-related problems or reactions (scale 0–4). In the analysis the total score was used. Data were available for 744 subjects.

### 2.3 Databases

Traffic insurance and police databases were used to obtain data about traffic collisions and traffic violations of the participants for the respective five-year period since the recruitment of each sample. For speeding violations and drunk driving (driving while intoxicated by alcohol - DWI) we also used the sum of violations in this five-year period, so that the score could be from 0 (no speeding tickets/drunk driving violations) to 5 (received at least one speeding/drunk driving violation ticket every year).

### 2.4 Genotyping

Genotyping for the triallelic classification of the 5-HTTLPR polymorphism was performed according to Anchordoquy et al. (2003). Genotyping was performed in two stages. First, all subjects were genotyped for the 5-HTTLPR VNTR polymorphism, then for the single nucleotide polymorphism (SNP) rs25531 (A/G). The polymorphic region was amplified using the primers 5-HTTLPR-F: 5′-6FAM-ATG CCA GCA CCT AAC CCC TAA TGT-3′ and 5-HTTLPR-R: 5′-GGA CCG CAA GGT GGG CGG GA-3′. Then SNP rs25531 (A→G) was determined as described in detail elsewhere (Tomson et al., 2011). Triallelic 5-HTTLPR genotypes were categorised into groups according to the effectiveness at the transcriptional level as follows: l_G_/l_G_, l_G_/s, and s/s were designated as s’/s’; l_A_/s and l_A_/l_G_ as l’/s’; and l_A_/l_A_ as l’/l’. Genotype frequencies were in the Hardy–Weinberg equilibrium. We compared the s’ allele carriers with the l’/l’ homozygotes (n ​= ​644, s’/l’ – 49.2%, l’/l’ – 32.0%, s'/s'– 18.8%).

### 2.5 Statistical analysis

Data were analysed using SPSS (version 23.0 SPSS, Chicago, IL) and SAS (version 9.4 SAS Inc., Cary, NC) software. Normality of data was assessed visually by box plots, Q-Q plots and also by skewness and kurtosis. Differences between groups regarding categorical variables were analysed with Pearson's chi-square test and the post-hoc Fisher's test, and for continuous variables with Student's t-tests if data was normally distributed and with Mann-Whitney *U* Test for data which did not follow normal distribution. Logistic regression analyses were used for predicting speed limit exceeding. First, simple logistic regression analyses were conducted with each independent variable, and next, independent variables were adjusted by gender.

To obtain insight into the complex relationship between traffic violations and accidents, genotype, impulsivity, eating and physical health behaviour, structural equation modelling (SEM) was used. For multiple imputation of missing cases Full Information Maximum Likelihood Estimation (FIML) was used. Fit indices and their acceptable thresholds used values as Root Mean Square Error of Approximation (RMSEA) less than 0.07 (Steiger, 2007), Comparative Fit Index (CFI) and Tucker Lewis index TLI greater than 0.95 (Sharma et al., 2005). The p ​< ​0.05 level was considered as statistically significant in all the analyses.

Correction for multiple testing was not used in this study.

## 3 Results

### 3.1 Health behaviour, impulsivity and aggressiveness

Demographic, health and traffic behavioural measures are presented in Table 1 separately for males and females. The males in our sample were significantly older than females. Males reported to eat less vegetables and had a higher body mass index, but regarding junk food consumption there was no significant gender difference. Males also had a higher AUDIT score and were to a greater extent engaged in vigorous physical activity per week. Regarding impulsivity and aggressiveness measures, males had higher scores in adaptive impulsivity (fast decision making and excitement seeking), but lower scores on the disinhibition subscale of maladaptive impulsivity. Males reported higher physical and verbal aggression, but females reported higher anger and hostility. The driving anger score was higher in males. In addition, males had more accidents and violations in traffic. With regard to high traffic risk (accidents ​+ ​violations), 65.7% of males had accidents and/or violations in the five-year period, vs 24.6% of females.

**Table 1:** Impulsivity and aggressiveness, health and traffic behaviour variables by gender.

	Females	Males	Total	Differences by gender
Age, mean (SD)	29.0 (8.0)	33.9 (11.1)	31.4 (10.0)	**t (725.8) ​= ​7.3, p ​< ​0.001**
Vegetable consumption, mean (SD)	5.02 (1.4)	4.53 (1.2)	4.78 (1.32)	**t (810.9) ​= ​-5.5, p ​< ​0.001**
Junk food consumption, median (min; max)	4 (2;8)	4 (2;8)	4 (2;8)	U ​= ​83051.0, p ​= ​0.957
Energy drink consumption (once a week/more) % (n)	7.0 (29)	10.5 (42)	8.7 (71)	χ^2^ ​= ​3.1, p ​= ​0.083
AUDIT score, median (min; max)	3 (0;20)	4 (0;31)	3 (0;31)	**U ​= ​47847.00, p ​< ​0.001**
Physical activity (hours per week), median (min; max)	1.2 (0;56.0)	3 (0;56.0)	2 (0;56.0)	**U ​= ​63344.0, p ​< ​0.001**
BMI (Body mass index), median (min; max)	22.7 (13.8;42.4)	26.3 (15.1;53.2)	24.5 (13.8;53.2)	**U ​= ​43252.0, p ​< ​0.001**
Fast decision making, mean (SD)	17.1 (4.6)	19.4 (4.5)	18.2 (4.6)	**t (772) ​= ​6.9, p ​< ​0.001**
Excitement seeking, mean (SD)	18.4 (5.1)	20.1 (5.2)	19.2 (5.2)	**t (772) ​= ​4.4, p ​< ​0.001**
Thoughtlessness, mean (SD)	15.3 (4.7)	14.7 (4.8)	15.0 (4.7)	t (772) ​= ​−1.8, p ​= ​0.075
Disinhibition, mean (SD)	17.7 (4.4)	16.8 (4.7)	17.3 (4.5)	**t (772) ​= ​-2.8, p ​= ​0.005**
Physical aggression (Buss-Perry), median (min; max)	13 (9;31)	15 (9;43)	14 (9;43)	**U ​= ​51443.5, p ​< ​0.001**
Verbal aggression (Buss-Perry), mean (SD)	12.2 (3.5)	13.1 (3.8)	12.6 (3.7)	**t (761) ​= ​3.4, p ​= ​0.001**
Anger (Buss-Perry), mean (SD)	15.0 (5.1)	13.6 (4.7)	14.3 (5.0)	**t (761) ​= ​-3.8, p ​< ​0.001**
Hostility (Buss-Perry), mean (SD)	17.2 (5.5)	16.3 (4.9)	16.7 (5.2)	**t (753.7) ​= ​-2.4, p ​= ​0.017**
Driving anger score (DAS 33), mean (SD)	52.9 (26.8)	61.1 (25.3)	57.4 (26.3)	**t (566) ​= ​3.8, p ​< ​0.001**
Traffic accidents (active), % (n)	12.0 (50)	23.1 (93)	17.5 (143)	**χ**^**2**^**= ​17.4, p ​< ​0.001**
Traffic accidents (passive), % (n)	8.4 (35)	23.9 (96)	16.0 (131)	**χ**^**2**^**= ​36.2, p ​< ​0.001**
Traffic accidents (all), % (n)	17.6 (73)	37.3 (150)	27.3 (223)	**χ**^**2**^**= ​40.0, p ​< ​0.001**
Traffic violations (speeding), % (n)	5.8 (24)	28.1 (113)	16.8 (137)	**χ**^**2**^**= ​72.9, p ​< ​0.001**
Traffic violations (DWI), % (n)	0.2 (1)	4.2 (17)	2.2 (18)	**χ**^**2**^**= ​15.1, p ​< ​0.001**
Traffic violations (all), % (n)	12.3 (51)	47.0 (189)	29.4 (240)	**χ**^**2**^**= ​118.7, p ​< ​0.001**
High traffic risk, % (n)	24.6 (101)	65.7 (249)	44.3 (350)	**χ**^**2**^**= ​117.9, p ​< ​0.001**

Significant differences are marked in bold; DWI – driving while impaired by alcohol; Means are presented for differences calculated by Student's t-test, medians for differences calculated by Mann-Whitney *U* test and percentages for differences calculated by Pearson's chi-square test and the post-hoc Fisher's test.

### 3.2 Health behaviour, impulsivity and aggressiveness in speed limit exceeders

Exceeding the speed limits is a most common traffic violation, and it was consistently associated with measures of impulsivity, aggressiveness and health behaviour (Table 2). Speed limit exceeders had higher scores of fast decision making (t (772) ​= ​−5.0, p ​< ​0.001) and excitement seeking (t (772) ​= ​−6.1, p ​< ​0.001), physical and verbal aggression (U ​= ​28899.0, p ​< ​0.001 and t (761) ​= ​−4.7, p ​< ​0.001, respectively) compared to subjects with no speeding tickets in the 5-year period, but they were not significantly different in maladaptive impulsivity (thoughtlessness and disinhibition), anger or hostility. Speeding subjects also had higher AUDIT score (U ​= ​32587.0, p ​= ​0.005), they reported doing more vigorous physical activity (U ​= ​34710.0, p ​< ​0.001) and drinking energy drinks more often (χ2 ​= ​7.21, p ​= ​0.007). Speed limit exceeders also had significantly more violations of other type, and higher prevalence of traffic accidents.

**Table 2:** Speed limit exceeding: Impulsivity and aggressiveness, health and traffic behaviour.

	Control group	Speed limit exceeders
Gender, male % (n)	42.5 (289)	82.5 (113)∗∗∗
Age, mean (SD)	31.2 (9.8)	32.4 (10.4)
Vegetable consumption, mean (SD)	4.80 (1.33)	4.69 (1.29)
Junk food consumption, median (min; max)	4 (2;8)	4 (2;8)
Energy drink consumption, once a week or more, % (n)	7.5 (51)	14.6 (20)∗∗
Alcohol problems (AUDIT), median (min; max)	3 (0;31)	4 (0;24)∗∗
Vigorous physical activity, hours per week, median (min; max)	2 (0;56)	3 (0;36)∗∗∗
BMI (Body mass index), median (min; max)	24.2 (13.8;47.2)	26.3 (15.1;53.2)∗∗
Fast decision making, mean (SD)	17.9 (4.6)	20.0 (4.5)∗∗∗
Excitement seeking, mean (SD)	18.8 (5.2)	21.7 (4.6)∗∗∗
Thoughtlessness, mean (SD)	14.9 (4.7)	15.3 (5.0)
Disinhibition, mean (SD)	17.2 (4.5)	17.4 (4.8)
Physical aggression (Buss-Perry), median (min; max)	13 (9;43)	16 (9;40)∗∗∗
Verbal aggression (Buss-Perry), mean (SD)	12.3 (3.6)	14.0 (4.0)∗∗∗
Anger (Buss-Perry), mean (SD)	14.1 (4.9)	15.0 (5.3)
Hostility (Buss-Perry), mean (SD)	16.7 (5.2)	16.8 (5.5)
Driving anger score (DAS 33), mean (SD)	55.7 (26.4)	64.6 (24.7)∗∗
Traffic accidents (all), % (n)	22.2 (151)	52.6 (72)∗∗∗
Traffic violations (DWI), % (n)	1.5 (10)	5.8 (8)∗∗
Traffic violations (other), % (n)	14.7 (100)	47.4 (65)∗∗∗

∗p ​< ​0.05, ∗∗p ​< ​0.01, ∗∗∗p ​< ​0.001 statistically significant difference. Means are presented for differences calculated by Student's t-test, medians for differences calculated by Mann-Whitney *U* test and percentages for differences calculated by Pearson's chi-square test and the post-hoc Fisher's test.

### 3.3 Gender differences in exceeding speed limits

We conducted logistic regression either unadjusted or adjusted for gender as there were significant gender differences in demographic and traffic behavioural variables (Table 1). Logistic regression, when adjusted for gender, rendered several of the previous significant associations with speeding, such as AUDIT score, BMI and DWI not statistically significant (Table 3). Several other associations remained significant even after adjustment, such as energy drink consumption, vigorous physical activity, adaptive impulsivity and aggression/anger scores as well as the frequency of traffic accidents occurrence.

**Table 3:** Logistic regression models predicting speed-limit exceeding.

	Speeding	Speeding^a^
Age	1.01 (0.99–1.03)	0.99 (0.97–1.01)
Gender, male vs female	**6.37 (4.00**–**10.15)**	-
Vegetable consumption, mean (SD)	0.94 (0.82–1.08)	1.06 (0.91–1.24)
Junk food consumption	1.18 (0.99–1.41)	1.18 (0.99–1.41)
Energy drink consumption, once a week vs less	**2.11 (1.21**–**3.66)**	**1.89 (1.05**–**3.40)**
Alcohol problems (AUDIT) score	**1.04 (1.01**–**1.08)**	1.00 (0.93–1.05)
Vigorous physical activity, hours per week	**1.04 (1.01**–**1.06)**	**1.03 (1.00**–**1.05)**
BMI (Body mass index)	**1.06 (1.02**–**1.09)**	1.01 (0.98–1.05)
Fast decision making score	**1.11 (1.06**–**1.16)**	**1.08 (1.03**–**1.13)**
Excitement seeking score	**1.13 (1.08**–**1.18)**	**1.11 (1.06**–**1.16)**
Thoughtlessness score	1.01 (0.98–1.06)	1.03 (0.99–1.07)
Disinhibition score	1.01 (0.97–1.05)	1.03 (0.99–1.07)
Physical aggression score (Buss-Perry)	**1.09 (1.05**–**1.12)**	**1.06 (1.02**–**1.10)**
Verbal aggression score (Buss-Perry)	**1.13 (1.07**–**1.19)**	**1.11 (1.05**–**1.17)**
Anger score (Buss-Perry)	1.03 (1.00–1.07)	**1.06 (1.02**–**1.11)**
Hostility score (Buss-Perry)	1.00 (0.97–1.04)	1.02 (0.98–1.06)
Driving anger score (DAS 33)	**1.01 (1.01**–**1.02)**	**1.01 (1.00**–**1.02)**
Traffic accidents all	**3.88 (2.65**–**5.68)**	**2.95 (1.98**–**4.40)**
Traffic violations (DWI), yes vs no	**4.16 (1.61**–**10.73)**	2.30 (0.87–6.04)
5-HTTLPR (n ​= ​644) La/La vs S allele	0.94 (0.61–1.45)	1.04 (0.77–1.41)

a adjusted for gender; Bold - significant predictor; odds ratio (OR) with 95 percent conﬁdence intervals (CI).

### 3.4 Drunk driving (DWI)

There were 18 subjects in the sample who had a drunk driving violation in the 5-year period, and 17 of them were male. So, when comparing drunk drivers with subjects who had no drunk driving violations, we included only male subjects (n ​= ​402; Table 4).

**Table 4:** Demographic and traffic behaviour variables in males by DWI.

Independent variable	No DWI (n ​= ​384)	DWI (n ​= ​17)
Age, mean (SD)	33.9 (11.2)	32.3 (9.3)
Vegetable consumption, mean (SD)	4.6 (1.2)	3.9 (1.2)∗
Junk food consumption, mean (SD)	3.9 (1.1)	4.2 (1.6)
Energy drink consumption, once a week or more, % (n)	10.4 (40)	11.8 (2)
Alcohol problems (AUDIT), median (min; max)	4 (0;31)	4 (3;31)∗
Vigorous physical activity, hours per week, median (min; max)	3 (0;56)	2 (0;30)
BMI (Body mass index), median (min; max)	26.3 (15.1;53.2)	26.0 (19.9;38.6)
Fast decision making, mean (SD)	19.4 (4.4)	18.2 (5.1)
Excitement seeking, mean (SD)	20.0 (5.2)	21.2 (6.8)
Thoughtlessness, mean (SD)	14.6 (4.8)	16.1 (5.0)
Disinhibition, mean (SD)	16.7 (4.7)	19.3 (4.3)∗
Physical aggression (Buss-Perry), median (min; max)	15 (9;43)	16 (10;40)
Verbal aggression (Buss-Perry), mean (SD)	13.0 (3.8)	14.5 (4.5)
Anger (Buss-Perry), mean (SD)	13.5 (4.6)	15.8 (6.4)
Hostility (Buss-Perry), mean (SD)	16.1 (4.8)	18.8 (5.1)∗
Driving anger score (DAS 33), mean (SD)	60.9 (25.5)	69.5 (15.0)
Traffic accidents (all), % (n)	23.9 (127)	23.5 (4)
Traffic violations (speeding), % (n)	27.3 (105)	47.1 (8)
Traffic violations (other), % (n)	32.5 (125)	70.6 (12)∗∗

∗p ​< ​0.05, ∗∗p ​< ​0.01, ∗∗∗p ​< ​0.001 statistically significant difference. Means are presented for differences calculated by Student's t-test, medians for differences calculated by Mann-Whitney *U* test and percentages for differences calculated by Pearson's chi-square test and the post-hoc Fisher's test.

Drunk drivers were significantly different by their higher disinhibition (t (381) ​= ​−2.2, p ​= ​0.029), an aspect of maladaptive impulsivity. Drunk drivers also had higher hostility (t (375) ​= ​−2.2, p ​= ​0.026) and significantly more other traffic violations (70.6% vs 32.5%, χ2 ​= ​10.53, p ​= ​0.001 compared to drivers with no DWI violation. Drunk drivers had higher AUDIT scores (mean rank ​= ​240.9 vs 181.2, U ​= ​2008.5, p ​= ​0.023) and they reported eating less vegetables than subjects with no drunk driving violation (t (399) ​= ​2.2, p ​= ​0.028).

### 3.5 Search for a path from the 5-HTTLPR to traffic violations

**Fig. 2:**
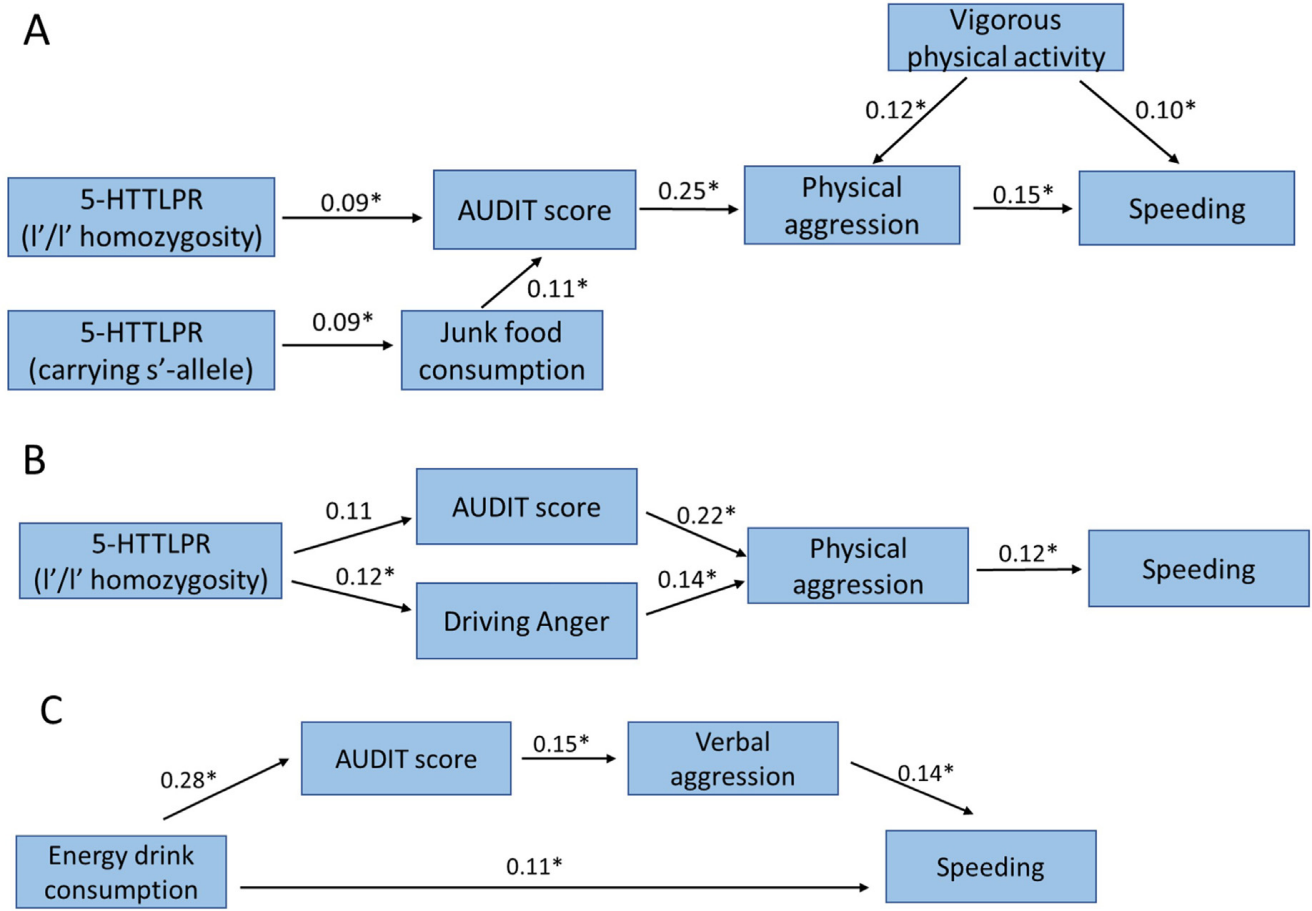
Path analysis models for exceeding speed limits. A – the whole sample (n ​= ​817), B - males (n ​= ​402), C - females (n ​= ​415).

In this sample neither drunk drivers nor speed limit exceeders differed by the frequencies of the 5-HTTLPR genotypes, but when we compared l’/l’ homozygotes with s’-allele carriers by other variables there were some differences: l’/l’ homozygotes had significantly higher AUDIT scores (mean rank ​= ​315.5 vs 285.8, U ​= ​33305.5, p ​= ​0.049) and reported eating less junk food (mean (SD) ​= ​3.7 (0.9) vs 3.9 (1.1), t (642) ​= ​−2.3, p ​= ​0.02), and additionally male l’/l’ homozygotes had higher driving anger scores as compared to s’-allele carriers (mean (SD) ​= ​64.1 (23.9) vs 57.3 (25.6), t (262) ​= ​2.0, p ​= ​0.04). Considering these differences and our hypothesis, we conducted structural equation modelling path analysis to uncover any indirect association of 5-HTTLPR with risky traffic behaviour and health behaviour measures. The results of this analysis suggested that those 5-HTTLPR l’/l’ homozygotes who have more problematic alcohol consumption and a higher score in physical aggression are more likely to speed in traffic (Fig. 2A). Those 5-HTTLPR s’-allele carriers who eat junk food more often join the path as consuming more alcohol and are more likely to speed in traffic. In addition, subjects who are more engaged in strenuous physical activity (sports/hard work) had increased physical aggression and speeding. All of the associations in the model were statistically significant (p ​< ​0.05), with a good fit to the model (RMSEA ​= ​0.009, CFI ​= ​0.99, TLI ​= ​0.99).

Considering the notable differences in risk-taking behaviour between males and females (Table 1, Table 3), path models for males and females were constructed separately. The path model for males again revealed l’/l’ homozygotes to have more speed limit exceeding mediated by AUDIT score and physical aggression, but also by driving anger with almost all of the associations in the model being significant with the exception of path from 5-HTTLPR to AUDIT score (p ​= ​0.056) (Fig. 2B; RMSEA ​= ​0.027, CFI ​= ​0.95, TLI ​= ​0.85).

Path analysis in females did not point at any role of the 5-HTTLPR, but energy drink consumption appeared as a significant predictor for speed limit exceeding on its own (Fig. 2C) as well as mediated through a higher AUDIT score and verbal aggression, and all of the associations in the model were statistically significant (RMSEA ​= ​0.000, CFI ​= ​1.00, TLI ​= ​1.11).

**Fig. 3:**
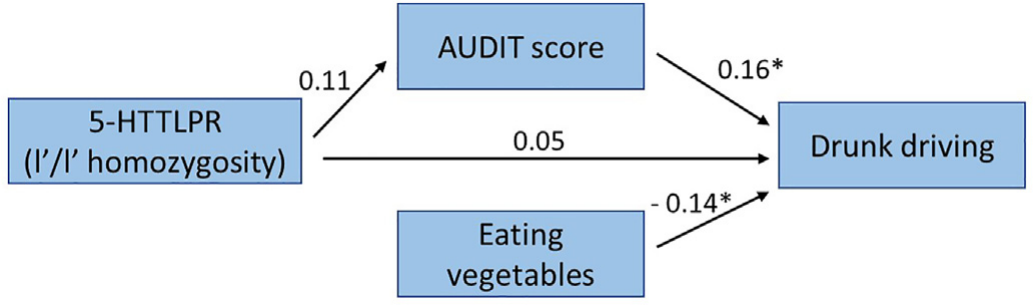
Path analysis model from 5-HTTLPR to drunk driving (males; n ​= ​402).

Path analysis for drunk driving also revealed an indirect association with l’/l’ homozygosity and drunk driving via higher AUDIT score (Fig. 3). In addition, eating less vegetables remained as a separate significant indicator of being a drunk driver. Although fit to the model was good, the paths from 5-HTTLPR to AUDIT score and 5-HTTLPR to drunk driving were not significant (p ​= ​0.055 and 0.390 respectively) (RMSEA ​= ​0.000, CFI ​= ​1.00, TLI ​= ​1.54).

## 4 Discussion

We have found that people who have higher impulsivity and/or aggressiveness violate traffic rules more often and also tend to be generally more reckless in their health behaviour. Exceeding speed limits was consistently associated with both personality factors and health behaviours. Speeding is a good example of a result of potentially impulsive decision-making in traffic and has possible dangerous outcomes (Elvik, 2012; Theofilatos and Yannis, 2014) and our data are consistent with speed limit exceeders having substantially more traffic accidents as compared with subjects who had no speeding tickets.

We have previously reported that speed limit exceeders have higher scores in facets of adaptive impulsivity (Paaver et al., 2006), and the present investigation extends this to aggressiveness. Higher aggressiveness among speeding subjects has also been shown before (Öz et al., 2010; Greaves and Ellison, 2011; Sârbescu and Rusu, 2021), and the use of Buss – Perry Aggression Questionnaire in the current study adds to this evidence with higher physical and verbal aggression of speed limit exceeders. Interestingly, speed limit exceeding was also associated with higher involvement in vigorous physical activity, both directly and mediated by physical aggression. Animal experiments has shown that exercise can effectively alleviate ADHD-like symptoms through enhancing dopamine D_2_ receptor expression in the brain (Sam Cho et al., 2014), and it has been suggested that exercise may serve as as a way of counteractive regulation of impulsive behaviours (Racine, 2012). It has also been shown that adults with ADHD engaging in frequent aerobic physical activity report significantly less behavioural impulsivity compared to subjects with low activity (Abramovitch et al., 2013). In addition, Joseph et al. (2011), have concluded that increased physical activity may help compensate and suppress the hedonic drive to over-eat. The results of this study do not suggest that speeding is a behaviour truly compensated for or suppressed by physical activity; rather exercising appears as being fuelled at least in part by similar mechanisms as the risky behaviour of speeding, and it is generally descriptive of the profile of someone with higher adaptive impulsivity.

Associations of lifestyle with aggression have also been shown before, with higher aggression levels among subjects with unhealthy lifestyles (sleeping less, poor eating habits, drinking, smoking, not working out) (Kim et al., 2020; Pouyamanesh, 2013; Rao et al., 2015). In a study using the Buss – Perry Aggression Questionnaire, the subjects with poor lifestyle had higher physical aggression, hostility, and anger, but those with healthier habits reported higher verbal aggression (Pouyamanesh, 2013). In the present study both physical and verbal aggression predicted speeding behaviour, but the former being a significant mediator in males and the latter in females. Even though we did not find a direct link between higher physical aggression, speeding and accidents in males, we did find that males had significantly more accidents in traffic as compared to females. Gender differences of physical vs verbal aggression in speeding behaviour appears consistent with previous studies which have found that females express their anger in traffic more constructively than males (González-Iglesias et al., 2012; Sullman, 2015; Hernández-Hernández et al., 2019). Thus, speeding in males and females is in part differently mediated and possibly for this reason more often resulting in accidents in males, because in traffic, higher physical aggression represents potentially more dangerous behavioural tendencies.

Overall, while speed limit exceeding was not directly associated with eating habits, drunk drivers reported eating less healthy foods by the example of vegetable consumption. Drunk drivers also had higher maladaptive impulsivity, as previously reported (Paaver et al., 2006), and aggressiveness measures. On the basis of previous studies on impulsivity and compulsive behaviour, we hypothesized that carriers of the 5-HTTLPR s’-allele would have higher maladaptive impulsivity and unhealthier eating habits, higher alcohol consumption and possibly also drunk driving, while 5-HTTLPR l’/l’ homozygotes might be prone to speeding. No simple association of the genotype with traffic behaviour was found. Admittedly the sample of subjects, especially with DWI, was small for this type of analysis. However, the path analysis models have suggested that the genotypes have both common and unique aspects in the path to traffic violations, contributing indirectly. The path of both l’/l’ homozygotes and s’-allele carriers leading to exceeding the speed limits included excessive use of alcohol and tendency of physical aggression, while the speeding s’-allele carriers also had less healthy dietary habits. A path specifically for the l’/l’ homozygotes leading through problematic alcohol use to drunk driving could also be established. It is obvious that both s’-allele carriers and l’/l’ homozygotes can exhibit both types of traffic violations, but speeding and drunk driving by l'/l’ homozygotes may occur in other contexts as of the s’-allele carriers, given their difference in aspects of impulsive and compulsive behaviour (Paaver et al., 2008; Walderhaug et al., 2007; Hong et al., 2018; Sinopoli et al., 2019). It should be noted that while drunk drivers in this study had higher frequency of other violations, they did not differ significantly in terms of involvement in traffic accidents. Further, in the path analysis with males, speeding among l’/l’ homozygotes was still associated with higher AUDIT scores, driving anger and physical aggression, but in females the association with energy drink consumption, higher AUDIT score and verbal aggression had no relationship to the 5-HTTLPR genotype. All together, the 5-HTTLPR l’/l’ homozygotes who had a record of either drunk driving or speeding were likely to be abusers of alcohol, and this was observable in males, although likely due to the decreased statistical power because of smaller sample size all of the associations were not separately significant. The 5-HTTLPR s’-allele carriers had a speeding record if they also presented further aspects of unhealthy lifestyle, here in the form of junk food eating. These findings are consistent with the view that the 5-HTTLPR l’/l’ homozygotes are behaviourally less flexible while the s’-allele carriers have higher sensitivity to the environmental context (Homberg and Lesch, 2011); on the other hand, behaviour of the 5-HTTLPR l’/l’ homozygotes may become more controlled by alcohol (Kapitau et al., 2019).

The previously reported association between consumption of energy drinks and high-risk behaviour (Hamilton et al., 2013) was also observable in the present study, and the association was stronger among those with higher AUDIT scores. That energy drink consumption is linked to high-risk behaviour particularly when combined with alcohol has also been observed (Breda et al., 2014). In traffic behaviour co-use of these beverages appeared particularly significant in females. Previously it has been found that high habitual caffeine consumers report greater trait-wise motor impulsivity, but acute caffeine intake did not influence response inhibition or impulsive, risky, or aggressive behaviour in high or low habitual caffeine consumers (Giles et al., 2016). So, it may be hypothesized that the consumption of energy drinks does not by itself induce risk-taking behaviour in traffic but is a behavioural tendency that accompanies those who take more risks.

As a limitation of the current study, it has to be pointed out that for a proportion of the subjects the data collection was carried out in different time windows. Impulsivity has been shown to decline steadily with age (Steinberg et al., 2008; Forrest et al., 2019), but the decline is more pronounced in the younger years, and it is unlikely that subjects of our study had a significant decline after the age of 30. Similarly, eating habits have been found to form already early during childhood (Nicklaus et al., 2005), and exercise behaviour to be moderately to highly stable across the life span (van der Zee et al., 2019). Therefore, it is also rather unlikely that the results of the study would have been affected substantially by this limitation of the database. It would have been desirable to know the diet in more detail, e.g., the intake of saturated fatty acids and sugar, but we doubted that it could be reliably reported by the participants. Therefore we focused on some of the most simple aspects of dietary choices which would reflect healthy and unhealthy behaviour.

## 5 Conclusion

In conclusion, several aspects of lifestyle like junk food consumption and physical activity were associated with taking risks and violating the rules in traffic. These maladaptive behavioural patterns appear to form around impulsive tendencies and develop along distinct patterns that in part vary by genetically encoded differences in neural circuits and include the gender factor. It might be beneficial to consider the constellations of the larger spectrum of health-related behaviours and the diversity of their mediating mechanisms in future interventions. This study expands our knowledge of the factors that are associated with behaviour in traffic and brings up research topics for the future, such as whether interventions that focus only on one behavioural aspect (e.g., traffic) should be converted into more comprehensive, and more personalized, approaches to lifestyle. Future interventions should, by means of promoting self-regulation skills, bring about a general change of behavioural habits in risk groups. The personalized aspect can be derived from understanding the genetic/biological variability that could inform about response to different types of intervention.

## Funding

This work was supported by the 10.13039/501100002301Estonian Research Council [grant number PRG1213], and also received funding from the European Union's Horizon 2020 research and innovation programme under grant agreement No 728018.

## Declaration of competing interest

The authors declare that they have no known competing financial interests or personal relationships that could have appeared to influence the work reported in this paper.
